# Green Extraction of Bioactive Compounds from Plant-Based Agri-Food Residues: Advances Toward Sustainable Valorization

**DOI:** 10.3390/plants14233597

**Published:** 2025-11-25

**Authors:** Samanta Shiraishi Kagueyam, José Rivaldo dos Santos Filho, Alex Graça Contato, Cristina Giatti Marques de Souza, Rafael Castoldi, Rúbia Carvalho Gomes Corrêa, Carlos Adam Conte Junior, Natália Ueda Yamaguchi, Adelar Bracht, Rosane Marina Peralta

**Affiliations:** 1 Post-Graduate Program in Food Science, State University of Maringá, Maringá 87020-900, PR, Brazil; samanta.ssk03@gmail.com; 2 Post-Graduate Program in Biochemistry, State University of Maringá, Maringá 87020-900, PR, Brazil; pg70637@uem.br (J.R.d.S.F.); cgmsouza@uem.br (C.G.M.d.S.); rcastoldi@uem.br (R.C.); abracht@uem.br (A.B.); 3 Food Analysis Center (NAL), Department of Biochemistry, Institute of Chemistry (IQ), Federal University of Rio de Janeiro (UFRJ), Rio de Janeiro 21941-598, RJ, Brazil; alexgraca.contato@gmail.com (A.G.C.); conte@iq.ufrj.br (C.A.C.J.); 4 Post-Graduate Program in Clean Technologies, Cesumar Institute of Science, Technology and Innovation—ICETI, Cesumar University, Maringá 87050-900, PR, Brazil; rubia.correa@unicesumar.edu.br; 5 Post-Graduate Program in Energy and Sustainability, Federal University of Santa Catarina, Araranguá 88905-120, SC, Brazil; natalia.ueda@ufsc.br; 6 Department of Chemical Engineering, State University of Maringá, Maringá 87020-900, PR, Brazil

**Keywords:** agri-food residues, bioactive compounds, green extraction, circular bioeconomy, sustainable nutrition, polyphenols, metabolic health, nutraceuticals, food waste valorization, biorefinery

## Abstract

Agri-food residues have accumulated globally at unprecedented scales, generating environmental pressures and resource inefficiencies, a core problem addressed in this review, while simultaneously representing rich, underutilized reservoirs of health-promoting phytochemicals. This review synthesizes recent advances (2016–2025) in the green extraction, characterization, and biological validation of phytochemicals from plant-based residues, including polyphenols, flavonoids, carotenoids, alkaloids, and dietary fibers from key sources such as grape pomace, citrus peels, coffee silverskin, pomegranate peel, cereal brans, and tropical fruit by-products. Emphasis is placed on sustainable extraction methods: ultrasound-assisted extraction (UAE), microwave-assisted extraction (MAE), pressurized liquid extraction (PLE), supercritical CO_2_ extraction (SFE), and natural deep eutectic solvents (NADES), which enable efficient recovery while minimizing environmental impact. In vitro, in vivo, and clinical studies demonstrate that residue-derived compounds exert antioxidant, anti-inflammatory, metabolic-regulating, and prebiotic effects, contributing to health in general and gut microbiota modulation. Integrating these bioactives into functional foods and nutraceuticals supports sustainable nutrition and circular bioeconomy goals by reducing food waste and promoting health-oriented valorization. Regulatory advances, including approvals from the European Food Safety Authority (EFSA) and the U.S. Food and Drug Administration (FDA) for ingredients such as olive phenolics, citrus flavanones, and coffee cascara, further illustrate increasing translational readiness. The convergence of green chemistry, biorefinery design, and nutritional science positions agri-food residues as pivotal resources for future health-promoting and environmentally responsible diets. Remaining challenges include scaling cost-effective green processes, harmonizing life cycle assessment protocols, expanding toxicological datasets, and conducting longer-term clinical trials to support safe and evidence-based commercialization.

## 1. Introduction

The rapid expansion of global food production and processing industries has resulted in an extraordinary volume of agricultural food residues. These by-products, such as peels, pomace, seeds, husks, shells, and bran, are often discarded despite being rich in specific high-value bioactive compounds, including hesperidin and naringin from citrus peels, resveratrol derivatives and anthocyanins from grape pomace, chlorogenic acids from coffee by-products, and lycopene from tomato pomace [1]. In the context of sustainability and circular bioeconomy, their valorization has become a strategic pathway toward waste minimization, environmental protection, and the creation of novel functional ingredients [2].

The circular economy paradigm has gained prominence in the food sector, emphasizing the need of “closing the loop” by reintegrating side streams into productive systems. Instead of linear “take–make–dispose” models, circular strategies promote the 7Rs: rethink, reduce, reuse, recycle, recover, renew, and redesign [3,4]. Within this framework, agri-food residues are now recognized as sustainable feedstocks for the extraction of bioactive molecules such as polyphenols, flavonoids, carotenoids, alkaloids, terpenoids, and dietary fibers, which include well-characterized compounds such as quercetin, catechins, gallic acid, β-carotene, mangiferin, and various bound phenolics that present strong antioxidant, anti-inflammatory, antimicrobial, and metabolic-regulating properties [5,6]. Bound phenolics refer to phenolic acids and related compounds covalently linked to cell-wall polysaccharides (e.g., hemicelluloses), requiring hydrolysis or enzymatic action for their release [7].

In recent years, there has also been a revolution in the techniques for extracting bioactive compounds from plant matrices. Conventional extraction techniques, such as maceration, Soxhlet extraction, or solvent percolation, are still widely used but have several drawbacks, including long processing times, high solvent consumption, and potential degradation of thermolabile compounds [8]. Emerging green extraction technologies, notably ultrasound-assisted extraction (UAE), microwave-assisted extraction (MAE), pressurized liquid extraction (PLE), and supercritical CO_2_ extraction (SFE), have been developed to overcome these limitations. These approaches rely on reduced solvent volumes, shorter processing times, and milder conditions, aligning with the principles of green chemistry and sustainability [5,9]. More recently, natural deep eutectic solvents (NADES) and enzyme-assisted extraction (EAE) have further expanded the technological toolbox for sustainably recovering polar phenolics, flavonoids, and other valuable metabolites [10]. Finally, apart from the laboratory scale, there is increasing interest in the industrial implementation of these extraction technologies and in integrating them within biorefinery schemes, allowing the sequential recovery of valuable molecules from a single feedstock. For example, grape pomace, one of the most studied residues, has been used to obtain polyphenols, fibers, and oils for nutraceutical and cosmetic applications [11,12,13,14]. Similarly, citrus peels, tomato pomace, coffee silverskin, pomegranate and onion peels, banana and mango peels, cocoa shells, and rice bran represent abundant global sources of phenolics and carotenoid compounds [15,16,17].

Considering these aspects, this review aims to provide a comprehensive examination of how bioactive compounds are obtained from agri-food residues through clean and sustainable extraction technologies. Specifically, it compiles recent evidence (2016–2025) on the main chemical classes recovered from diverse agro-industrial by-products and summarizes their demonstrated biological activities in both in vitro and in vivo models. In addition, the review analyzes current technological innovations, industrial applications, life-cycle implications, commercial developments, and intellectual property trends, emphasizing how green extraction strategies contribute to circular bioeconomy frameworks.

## 2. Methodology

Structured searches were conducted in Scopus, Web of Science, PubMed, and Google Scholar between January 2016 and March 2025 using combinations of keywords (e.g., agri-food residues, green extraction, grape pomace, citrus peels, coffee by-products, UAE, MAE, SFE, NADES, bioactive compounds, in vitro, in vivo, clinical trial). The residues were selected in this review based on three principal criteria: (i) high global production volumes and industrial relevance; (ii) availability of recent (2016–2025) studies describing green extraction and chemical characterization; and (iii) the existence of in vitro, in vivo, or clinical evidence supporting their biological effects. Other aspects that were considered were industrial applications, life cycle aspects, the existence of commercial products, and intellectual property (patents) that demonstrate the real potential of residue valorization in the framework of a circular bioeconomy. Only peer-reviewed articles in English were included, while conference abstracts, non-scientific documents, or studies mixing food residues with non-food waste streams were excluded. Although the review is narrative, a structured search ensures consistency and comprehensive coverage.

## 3. Bioactive Compounds

The chemical composition of agri-food residues is highly variable, depending on botanical origin, anatomical part, and processing method. Nonetheless, they consistently contain bioactive molecules with recognized functional and biological relevance. Major chemical classes include polyphenols, flavonoids, tannins, phenolic acids, anthocyanins, carotenoids, terpenes, alkaloids, fatty acids, and polysaccharides, many of which contribute to antioxidant capacity and health-promoting effects [6,9]. Globally abundant residues such as grape pomace (>10 million tons/year), citrus peels (>15 million tons/year), coffee by-products (silverskin, pulp, husk), cereal brans, and tomato pomace represent some of the most productive sources for bioactive recovery [18,19].

Polyphenols are among the most abundant phytochemicals in agri-food residues. They include flavonoids (quercetin, kaempferol, catechins), phenolic acids (gallic, caffeic, ferulic, p-coumaric), stilbenes (resveratrol), and ellagitannins (punicalagin). Industrial residues such as grape pomace, pomegranate peel, coffee silverskin, onion skins, peanut skins, and apple pomace are rich sources of these compounds. Numerous in vitro and in vivo studies demonstrate their antioxidant, anti-inflammatory, antidiabetic, and anti-obesity activities [14,20,21]. Ellagitannins from pomegranate peels, for example, are microbially converted into urolithins, metabolites with recognized anti-inflammatory and mitochondrial-modulating effects, while catechins from cocoa shells and tea residues enhance antioxidant defenses and inhibit microbial growth [22,23,24].

Carotenoid-rich residues such as tomato pomace, mango peel, and carrot processing by-products contain lycopene, β-carotene, and lutein, compounds with antioxidant and antiproliferative properties. Advances in SFE, pressurized ethanol, MAE, and enzyme-assisted approaches have increased yields and purity while reducing solvent use [25]. Tomato pomace, with lycopene levels ranging from 250 to 1000 mg/kg dry matter, is especially valuable for producing natural colorants and lipid-protective ingredients. Recent studies show significantly enhanced carotenoid recovery from tomato residues using microwave-assisted and high-pressure extraction [26,27].

Fiber-rich residues, including banana peels, brewers’ spent grain (BSG), rice bran, and citrus peel, contain both insoluble fibers and bound phenolics that enhance antioxidant and prebiotic potential. Green extraction strategies frequently combine enzymatic or alkaline hydrolysis with UAE to release conjugated phenolic fractions [28,29]. Banana peel pectin, for instance, exhibits gel-forming behavior and contributes to lipid-lowering effects, while ferulates extracted from cereal brans show anti-inflammatory and gut-barrier–strengthening activities [29,30]. In addition, residues such as cocoa shells and mango peel contain bioactive methylxanthines (theobromine, caffeine) and xanthones (mangiferin), which contribute to metabolic regulation, antioxidant defenses, and antimicrobial properties [31,32]. These molecules extend residue applications beyond food supplements, enabling their incorporation into cosmeceutical, pharmaceutical, and oral-care formulations. Cocoa shell extracts rich in methylxanthines and phenolics have been tested in antimicrobial oral-care prototypes, while mangiferin-rich mango peel extracts show α-glucosidase inhibitory activity and glycemic control potential [33,34].

Altogether, the high density of polyphenols, carotenoids, fibers, methylxanthines, and other phytochemicals in these residues highlights their biological relevance and strengthens their role in sustainability-oriented value chains. Because these compounds occur in concentrated amounts relative to the original biomass, their recovery enables substantial value addition, reduces environmental burdens associated with disposal, and supports circular bioeconomy models in which side streams are transformed into functional food ingredients, nutraceuticals, cosmetics, or biomaterial precursors. This link between chemical richness and circularity is a driving force behind the growing global interest in residue valorization [35,36,37].

## 4. Green Extraction Technologies

The transition from conventional extractive approaches to green extraction technologies is an essential requirement for the sustainability of agro-food residue biorefineries. Green extraction technologies, such as UAE, MAE, PLE, SFE, NADES and EAE, are based on solvent reduction and increased selectivity, complying with the 12 Principles of Green Chemistry [5,9,38].

Green extraction strategies emphasize efficiency, selectivity, and preservation of thermolabile compounds while reducing solvent and energy use. Comparative studies show that replacing maceration or Soxhlet extraction with green processes can reduce solvent consumption by 40–90% and extraction times by up to 80% [39].

### 4.1. Ultrasound-Assisted Extraction (UAE)

UAE employs acoustic cavitation (20–100 kHz) to generate microbubble formation and collapse, disrupting cell walls and increasing solvent penetration. It is characterized by low energy demand, short extraction times, and excellent compatibility with aqueous–ethanolic solvents. UAE is widely applied to extract polyphenols from grape pomace, ferulic acid from brewer’s spent grain, and pigments from onion skins [11,28,40,41]. It can increase the total phenolic yield by 20–45% and reduce energy consumption by ~35% [42]. Because of its tissue-disruption action, UAE is also an effective pre-treatment for MAE or EAE.

### 4.2. Microwave-Assisted Extraction (MAE)

MAE uses dielectric heating to promote rapid molecular rotation in polar matrices, leading to internal heating, cell rupture, and accelerated solute diffusion. MAE is especially efficient for extracting phenolics, flavonoids, and alkaloids from citrus residues, pomegranate peel, grape pomace, and tropical by-products [2,8]. It increases phenolic recovery by 20–100% and reduces extraction times from hours to minutes [43]. Continuous-flow MAE systems have recently improved heating uniformity and scalability [44]. MAE is particularly suited to residues rich in polar or tightly bound phenolic fractions.

### 4.3. Pressurized Liquid Extraction (PLE)

PLE (also known as ASE) applies elevated pressures (10–15 MPa) and moderate temperatures (50–200 °C) to increase solvent diffusivity and maintain it in the liquid state. It efficiently recovers phenolic acids, flavonoids, and carotenoids from tomato pomace, coffee by-products, and cereal brans [5,45]. PLE can increase carotenoid yields by 30–65% and reduce solvent consumption by >70% compared to Soxhlet [46]. It is commonly integrated into multi-step biorefinery workflows following UAE or MAE.

### 4.4. Supercritical CO_2_ Extraction (SFE)

SFE uses CO_2_ above its critical point (≥31 °C, ≥7.38 MPa), achieving gas-like diffusivity and liquid-like solvating power. It is ideal for extracting nonpolar and moderately polar compounds such as carotenoids, sterols, and lipophilic phenolics. Several studies report multiple-fold increases in carotenoid concentration for supercritical CO_2_ + co-solvent compared to conventional extraction [47]. Ethanol (5–15%) is often used as a co-solvent to extract semi-polar compounds. Although capital costs are high, continuous-flow SFE systems have improved energy efficiency and scalability [48].

### 4.5. Natural Deep Eutectic Solvents (NADES)

NADES, biodegradable liquids formed from sugars, organic acids, and amino acids, have been increasingly employed as tunable-polarity extraction media capable of selectively recovering phenolics, flavonoids, alkaloids, and other polar metabolites. Studies on agri-food residues such as pomegranate peel and grape pomace have demonstrated that NADES generally yield higher polyphenol recoveries than aqueous ethanol, although the magnitude of this increase varies depending on the specific solvent system and biomass matrix [49]. Challenges remain regarding high viscosity and difficult solvent regeneration; however, recent advances in membrane-based separation, pervaporation, and vacuum-assisted evaporation have significantly improved NADES recyclability, with several studies reporting high recovery efficiencies under optimized conditions [50].

### 4.6. Enzyme-Assisted Extraction (EAE)

EAE employs enzymes such as cellulases, pectinases, xylanases, and feruloyl esterases to degrade polysaccharide matrices and release bound bioactives. It enhances extraction under mild conditions and reduces the need for chemical solvents. EAE increases the release of bound phenolics from cereal brans and coffee by-products by 30–70% [51]. Combining EAE with UAE or PLE improves mass transfer and extraction efficiency while supporting clean-label production goals.

To address the reviewer’s request for clearer organization, Table 1 summarizes each green extraction method, residue type, major bioactive class recovered, and their key operational advantages, providing a visual complement to the narrative.

## 5. In Vitro Bioactivity Evidence for Agri-Food Residues

Numerous in vitro studies have been conducted to evaluate the functional potential of bioactive compounds recovered from agri-food residues. These experiments provide mechanistic insights into antioxidant, antimicrobial, anti-inflammatory, enzymatic, and prebiotic functions. The outcomes highlight that different residues, depending on their chemical composition and extraction method, yield distinct biological responses. Polyphenol-rich extracts from grape pomace, pomegranate peel, and onion skins exhibit strong radical-scavenging and anti-inflammatory effects, whereas cocoa shells, banana peels, and tomato pomace show promising antioxidant and antimicrobial properties. Fiber-rich by-products such as brewer’s spent grain (BSG) and coffee silverskin demonstrate additional prebiotic potential, enhancing the circular value of these residues in food systems [14,21,28].

In vitro assays commonly evaluate endpoints related to oxidative stress, including inhibition of reactive oxygen species (ROS) and reduction in lipid peroxidation. Extracts from pomegranate peel and coffee silverskin, for example, significantly attenuate ROS accumulation and lipid-peroxidation processes in intestinal and macrophage cell models [21,52]. Onion-skin flavonol extracts also demonstrate potent radical-scavenging activity attributed to quercetin derivatives [53].

Suppression of pro-inflammatory mediators such as nitric oxide (NO), IL-6, and TNF-α is another widely documented response. Pomegranate peel polyphenols have shown significant inhibitory effects on NO and pro-inflammatory cytokines, while similar anti-inflammatory actions have been observed in vitro for coffee silverskin phenolics [54,55].

Residue-derived phenolics also play a role in metabolic regulation through inhibition of α-amylase and α-glucosidase, key enzymes involved in postprandial glucose release. Phenolic extracts obtained from grape, citrus, apple, and mixed fruit residues exhibit substantial inhibitory activity against these enzymes, reflecting their potential for glycemic control [56,57,58]. These effects are attributed to flavonoids, hydroxycinnamic acids, and tannins capable of interacting with enzymatic active sites.

Prebiotic responses are likewise frequently observed in vitro. Coffee silverskin extracts enhance the growth of probiotic bacteria such as *Lactobacillus* and *Bifidobacterium* and stimulate short-chain fatty acid (SCFA) production during fermentation assays [59]. BSG- and fruit-peel-derived fibers similarly promote SCFA formation and microbial metabolic activity, confirming their role as fermentable substrates [60,61]. Table 2 summarizes the in vitro evidence on recovered compounds organized by chemical class, residue type, extraction strategy, and reported bioactivities. These in vitro investigations confirm that residues are reliable sources of functional molecules with multiple biological activities. Importantly, the studies also demonstrate that green extraction methods can achieve yields comparable to conventional extractions while substantially lowering environmental impact [2]. Collectively, the antioxidant, anti-inflammatory, enzyme-inhibitory, and prebiotic mechanisms observed across different residue sources reinforce their potential for incorporation into functional foods, nutraceuticals, and microbiota-targeted formulations. These functional responses align with circular bioeconomy goals by transforming agri-food residues into value-added, health-promoting ingredients. The in vitro evidence supports further in vivo and clinical studies aiming to validate efficacy and safety in biological systems.

## 6. In Vivo Bioactivity Evidence for Agri-Food Residues

Animal and human studies provide translational evidence for residue-derived bioactives, complementing in vitro findings and clarifying dose–response, bioavailability, and safety. Dietary interventions with grape pomace polyphenols consistently reduce weight gain and improve short-chain fatty acids (SCFAs) and microbiota composition in high-fat diet mice [68]. Pomegranate peel ellagitannins (punicalagin, ellagic acid → urolithins) modulate inflammasome signaling (NLRP3/caspase-1/IL-1β), improving cardiometabolic endpoints in diabetic rats [69]. Hydroxytyrosol (HT) from olive side streams improves antioxidant/anti-inflammatory status in overweight or prediabetic adults [70]. Citrus peel flavanones (hesperidin, naringin) show benefits on lipids, blood pressure, and glycemic markers across randomized trials [71,72]. Prebiotic effects are recurrent for coffee silverskin, increasing SCFAs and improving selected metabolic readouts in rodent models [59]. Additional streams (banana peel pectin, mango peel mangiferin, peanut skin proanthocyanidins, cashew apple bagasse, and apple pomace polyphenols) demonstrate anti-obesity, gut-barrier, anti-inflammatory, and neuroprotective signals in vivo [13,29,31,73,74].

For instance, mango peel polyphenols improve hepatic antioxidant defenses and reduce lipid peroxidation in diabetic models [75], while pomegranate-peel urolithin metabolites enhance mitochondrial function and intestinal barrier integrity in vivo [76]. These mechanistic improvements complement systemic metabolic outcomes and reinforce the biological plausibility of residue-derived interventions. Furthermore, emerging evidence suggests that agri-food residue extracts may modulate gut–brain and gut–liver axes. Apple pomace polyphenols, for example, attenuate neuroinflammation and improve hippocampal signaling pathways involved in memory processes [74]. Together, these studies support the circular-bioeconomy proposition that residue streams can supply clinically relevant bioactives, especially when combined with standardized extraction protocols, dose normalization, and chemically characterized phenolic profiles that enable improved reproducibility and regulatory acceptance. Ensuring alignment with established safety parameters is essential for translating these bioactives into human applications.

## 7. Critical Appraisal and Translational Considerations

In vivo evidence summarized in Table 3 reveals that phenolic- and fiber-rich extracts from grape pomace, pomegranate peel, citrus peel, olive residues, coffee silverskin, and other agri-food by-products consistently produce antioxidant, anti-inflammatory, metabolic, and gut-modulating effects in rodent models. These converging outcomes support the translational potential of residue-derived compounds as functional ingredients and nutraceuticals, provided that dosing, standardization, and regulatory safety are properly addressed.

As shown in Table 3, the in vivo studies generally employ extract doses between 100 and 500 mg·kg^−1^·day^−1^. There are two exceptions, however, in which much higher doses were used, namely 1 g·kg^−1^ for coffee silverskin and 5 g·kg^−1^ for mango peel. Table 3 also informs about the possible human-equivalent doses calculated using a translation formula based on surface area [77]. For daily rodent doses of up to 500 mg/kg, the hypothetical human dose (2.83 g for a 70 kg individual) is still feasible in the context of a crude preparation. For coffee by-products and mango peel preparations, however, the equivalent human doses would be impractically high, suggesting the need for standardization and purification strategies that enrich specific bioactive markers.

To achieve clinical relevance, extracts must be standardized to specific compounds such as punicalagin in pomegranate peel [69], HT in olive residues [70], or hesperidin in citrus peel [71], rather than described broadly as “polyphenol-rich.” Lack of chemical fingerprinting limits reproducibility, regulatory acceptance, and evidence-based health-claim substantiation. Several phenolics listed in Table 3 undergo extensive phase II conjugation (glucuronidation, sulfation, methylation) and microbial conversion into bioactive metabolites such as urolithins derived from ellagitannins. This process explains the variable efficacy observed among studies and highlights the influence of gut microbiota composition on response magnitude.

Recent findings indicate that the metabolic fate of residue-derived compounds can meaningfully affect therapeutic outcomes. For example, urolithin A generated from ellagitannins improves mitochondrial function and intestinal barrier integrity in vivo [78] underscoring the importance of microbial biotransformation in the biological activity of residue-derived phenolics. Similarly, mango peel polyphenols enhance hepatic antioxidant systems and reduce lipid peroxidation in diabetic animals [75]. These mechanisms reinforce the need for studies that integrate both systemic and tissue-level biomarkers. Personalized nutrition and symbiotic formulations, combining fiber matrices from residues (e.g., coffee silverskin or banana peel) with phenolics, represent a promising approach to enhance both bioavailability and colonic transformation [66]. Such combined strategies may potentiate microbial fermentation, promote SCFA formation, and improve phenolic metabolite profiles, supporting more consistent clinical responses across different microbiota phenotypes.

**Table 3:** In vivo evidence (2016–2025) in animals (rats and mice) for bioactives from agri-food residues, with models, principal outcomes, green extraction, intended application, and circularity notes reduction.

Chemical Class/Identified Bioactives	Residue (Origin) and Circularity/LCA Notes/Green Extraction	Hypothetical Human Dose Calculated According to a Translation Formula Based on Surface Area [77]	In Vivo Model and Outcomes/Intended Application	Ref.
Polyphenol mixture (anthocyanins, flavanols, phenolic acids. Catechins, quercetin, gallic/caffeic acids, procyanidins	400 mg/kg body weight grape pomace (wine coproduct)—upcycled ingredient; valorizes winery waste; potential greenhouse gas reduction. Hydroethanolic extraction; spray-dry	32 mg/kg; human dose for a 70 kg individual = 2.24 g	High-fat diet mice: reduction of body-weight gain; increase short-chain fatty acids; improved microbiota composition. Weight-management functional ingredient	[68]
Ellagitannins and derivatives. Punicalagin, ellagic acid → urolithins	150 mg/kg body weight pomegranate peel (juice waste)—cardiometabolic protection. High-phenolic density from peel; biorefinery node. Hydroethanolic extraction; purification	24 mg/kg; human dose for a 70 kg individual = 1.68 g	Diabetic rats: reduction NLRP3/caspase-1/IL-1β; improved lipid profile; histological protection. Cardiometabolic nutraceutical	[69]
Phenolics and fiber (coffee by-product). Chlorogenic acids (minor), fiber-bound oligosaccharides	1 g/kg body weight coffee silverskin (roasting by-product) prebiotic. Avoid landfilling/incineration of silverskin. Green UAE; low-energy drying	162 mg/kg; human dose for a 70 kg individual = 11.34 g	Rats: ↑ SCFAs; improved metabolic readouts; microbiota shifts. Prebiotic ingredient	[59]
Polyphenol mixture (apple). Chlorogenic acid, phloridzin, quercetin glycosides	100 mg/kg body weight apple pomace (juice/cider residue) neuroprotective candidate. Pomace biorefinery (polyphenols + pectin). Aqueous ethanol extraction; stabilization	8.1 mg/kg; human dose for a 70 kg individual = 0.57 g	Mice: reversal of MK-801-induced memory impairment; hippocampal gene modulation. Cognitive-health dietary ingredient	[74]
Soluble fibers (pectin). High-methoxyl pectin; minor phenolics	10% diet banana peels (fruit processing waste) anti-obesity fiber. Supports zero-waste in banana chain. Hot-water extraction; ethanol precipitation	-	Obese hypercholesterolemic mice: improved adiposity and lipid profile. Fiber supplement; fat-reduction aid	[29]
Xanthones and phenolics. Mangiferin; quercetin derivatives	5 g/kg body weight mango peel (juice/drying waste)—standardized extract. Revenue from peels complements fruit value chain. Hydroethanolic extraction; standardization	810.8 mg/kg; human dose for a 70 kg individual = 56.8 g	Prediabetic rats: improved glycemia and lipids; enzyme inhibition (α-amylase/α-glucosidase)/Metabolic-health nutraceutical	[31]
Proantho-cyanidins (A-type). Procyanidin A1	300 mg/kg body weight peanut skins (blanching waste)—polyphenol extract. Requires allergen controls in scale-up. Ethanolic extraction; enrichment	24 mg/kg; human dose for a 70 kg individual = 1.68 g	Type 2 diabetes mice: improved gut barrier (tight junctions); anti-inflammatory effects/Gut-barrier/anti-inflammatory nutraceutical	[79]
Mixed phenolics. Anacardic acids, carotenoids, phenolic acids	500 mg/kg body weight cashew apple bagasse (juice residue)/standardized extract. Valorizes bagasse in cashew processing. Hydroethanolic extraction; spray-dry	40.5 mg/kg; human dose for a 70 kg individual = 2.83 g	DSS-colitis in mice: improved disease activity. immunomodulatory protection. Anti-inflammatory (preclinical inflammatory bowel disease)	[73]

Translation of residue-derived extracts into marketable products requires alignment with existing food-safety and novel-food frameworks. Within the European Union (EU), olive-oil phenolics (notably HT) are authorized for a health claim under Regulation No. 432/2012 [80], while citrus fiber is classified as Generally Recognized as Safe (GRAS) by the U.S. Food and Drug Administration (FDA) under Notice No. 943 [81]. The European Food Safety Authority (EFSA) recently approved dried coffee husk (cascara) as a novel food, illustrating increasing regulatory openness to circular valorization. Nonetheless, new ingredients such as mango peel or peanut-skin extract still require toxicological substantiation, allergenicity assessment, and compositional standardization before approval in jurisdictions including EFSA, FDA, or Brazil’s National Health Surveillance Agency (ANVISA) [82].

Advances in analytical chemistry, particularly high-resolution HPLC–MS fingerprinting, have improved the ability to characterize complex residue-derived extracts and ensure batch-to-batch consistency, which is critical for safety dossiers and regulatory filings [83]. Validated analytical workflows are essential for identifying marker compounds, detecting contaminants, and establishing safe intake ranges.

Among the bioactives cataloged in Table 4, the most robust human evidence concerns olive phenolics and citrus flavanones, both supported by randomized controlled trials [70,71]. For other residues, including grape pomace, coffee silverskin, peanut skins, mango peel, and banana peel, clinical validation remains limited. For grape pomace, a crossover clinical trial provides preliminary human data [84], but sample sizes remain small. For coffee silverskin, peanut skins, mango peels, and banana peels, existing evidence largely consists of reviews, in vitro and rodent studies, or pilot trials, indicating a need for more rigorous human intervention studies [85,86].

Future research should prioritize double-blind, placebo-controlled trials employing standardized extracts, validated biomarkers (e.g., inflammatory cytokines, lipid profile, SCFAs), and controlled dietary designs to clarify dose–response relationships and real-world efficacy. Integrating LCA and toxicological endpoints into these trials will further strengthen the sustainability and safety dimensions of residue-derived functional ingredients, positioning them as credible components of circular nutrition and public health strategies.

## 8. Implications of Valorizing Agri-Food Residues for Bioactive Recovery or Human Nutrition and Health

Using agri-food residues to obtain bioactive compounds offers a sustainable way to enhance diet quality and promote metabolic and intestinal health. Among residue-derived compounds, olive phenolics and citrus flavanones currently have the strongest human evidence, with growing, though still emerging, signals for coffee by-products (cascara, silverskin) and ellagitannin-rich streams (e.g., pomegranate peel) via their gut-microbial metabolites.

Randomized trials now indicate that HT from olive side streams can improve redox and inflammatory biomarkers in adults with overweight or prediabetes when provided as a standardized extract over 16 weeks, supporting cardiometabolic risk-reduction strategies and aligning with existing EU authorization for olive-oil phenolic claims (≥5 mg/day) when adequately standardized [70]. Meta-analyses and systematic reviews likewise suggest that hesperidin, a citrus-peel flavanone recoverable in biorefinery cascades, can lower fasting glucose and atherogenic lipids, with stronger effects at >500 mg/day and >12 weeks, informing pragmatic dosing windows for clinical translation [71,87]. Together, these findings highlight two core translational levers for residue-derived nutraceuticals: quantified marker compounds (e.g., HT, hesperidin) and exposure sufficiency (dose × duration) concordant with trial evidence.

Several residue-derived phenolics act through gut-microbiota transformation, generating metabolites with distinct pharmacokinetics and bioactivities. Ellagitannins from pomegranate peel are converted to urolithins, which engage mitochondrial and anti-inflammatory pathways; inter-individual urolithin metabotypes help to explain heterogeneous responses and support microbiome-aware personalization in future trials and functional-food design [88,89]. Recent human and preclinical studies show that urolithin A supplementation improves mitochondrial efficiency, gut-barrier integrity, and inflammatory biomarkers, reinforcing its relevance as a downstream metabolite of residue-derived ellagitannins [90].

Formulation strategies that co-deliver fiber matrices derived from residue streams (e.g., coffee silverskin) along with phenolic traces (e.g., chlorogenic acids) stimulate probiotic growth and organic-acid production in colon-relevant models [66]. Such symbiotic combinations enhance SCFA formation and improve metabolic markers, supporting integrated approaches that combine the fermentability of fibers with the bioactivity of phenolics [91].

Policy shifts are beginning to shorten the path from residue to diet. The EFSA novel-food authorization for dried coffee husk (cascara) enables its use in beverages and foods, creating a regulated on-ramp for coffee by-product polyphenols and fibers. While human trials on coffee silverskin remain limited, preclinical and in vitro data support prebiotic and antioxidant roles, warranting well-designed randomized controlled trials (RCTs) using standardized materials now that regulatory frameworks exist to support market entry [92]. Standardized extraction protocols and HPLC-MS–based fingerprinting have recently improved quality-control metrics for upcycled botanical ingredients, facilitating regulatory acceptance [93].

For incorporation into foods, medical foods, or supplements, residue-derived bioactives benefit from delivery systems that enhance stability and bioaccessibility, such as microencapsulation for hesperidin or emulsions and sustained-release matrices for HT. Given the dose–duration thresholds seen in clinical trials, food formats that enable consistent daily intake (e.g., beverages, yogurts, bars) may be preferable to sporadic supplement use [94]. Co-formulation with compatible fibers (citrus, cereal, coffee silverskin) may improve metabolic outcomes by coupling prebiotic and antioxidant pathways while supporting clean-label claims [95]. Emerging food-matrix studies demonstrate that phenolic–fiber interactions can increase the bioaccessibility of flavanones and phenolic acids during digestion [96].

Safety dossiers for upcycled ingredients (e.g., cascara) demonstrate a maturing regulatory ecosystem; however, broader toxicological profiling, allergenicity assessment, and marker-based quality control remain necessary for many residue extracts. Priority areas for nutrition-aligned research include: (i) multi-arm RCTs comparing standardized residue-derived extracts at graded doses and ≥12-week exposures; (ii) metabotype-stratified analyses (e.g., urolithin producers vs. non-producers); (iii) food-based delivery trials that track validated endpoints (lipids, glycemia, blood pressure, inflammatory cytokines, SCFAs) alongside adherence and tolerability; and (iv) bioequivalence comparisons between upcycled-ingredient sources and conventional botanicals [97].

Summarizing, residue-derived bioactives, especially olive phenolics and citrus flavanones, already meet several criteria for nutritional relevance, including identifiable marker compounds, reproducible human evidence, and emerging regulatory recognition. As microbiome-aware designs, long-duration clinical trials, and food-format delivery systems advance, agri-food residues can transition from underutilized side streams to scientifically validated components of cardiometabolic nutrition [98].

## 9. Circularity, Applications, and Life Cycle Assessment (LCA) Perspectives

Valorizing agri-food residues as sources of bioactives delivers environmental, technological, and socioeconomic benefits when recovery processes are structured as biorefinery cascades and evaluated under life cycle thinking. Agri-food residues, often considered waste, are in fact rich sources of valuable compounds. In cascading valorization, residues are reinterpreted as renewable feedstocks that can be sequentially processed, with each extraction step generating a product stream while leaving behind a substrate suitable for further transformation. This hierarchical design ensures full resource utilization, prioritizing high value bioactives such as polyphenols, carotenoids, and xanthones [99], followed by recovery of pectins, fibers, proteins, and lipids for food, feed, biomaterial, or biopolymer applications [100]. Residual fractions can then be directed to anaerobic digestion or composting to close the loop through renewable energy generation or soil nutrient replenishment [101,102].

Recent LCA evidence demonstrates that multi-output biorefinery cascades significantly outperform single-product valorization. For grape pomace, citrus peel, and coffee residues, integrated extraction–fiber recovery–bioenergy models reduce greenhouse-gas emissions by 35–60%, depending on the allocation method and solvent recycling efficiency [100]. For coffee by-products, combining polyphenol extraction with anaerobic digestion or composting results in markedly lower cumulative energy demand and eutrophication potential compared with direct disposal [103]. Extraction technology is a critical driver of environmental performance. LCA comparisons show that green extraction techniques, UAE, MAE, SFE, and NADES, achieve substantially lower environmental footprints than conventional solvent extraction due to reduced thermal loads, shorter processing times, and minimal solvent losses [104]. These advantages become even more pronounced when extracts are destined for food applications where purity requirements are high and downstream processing can be minimized.

**Figure 1:**
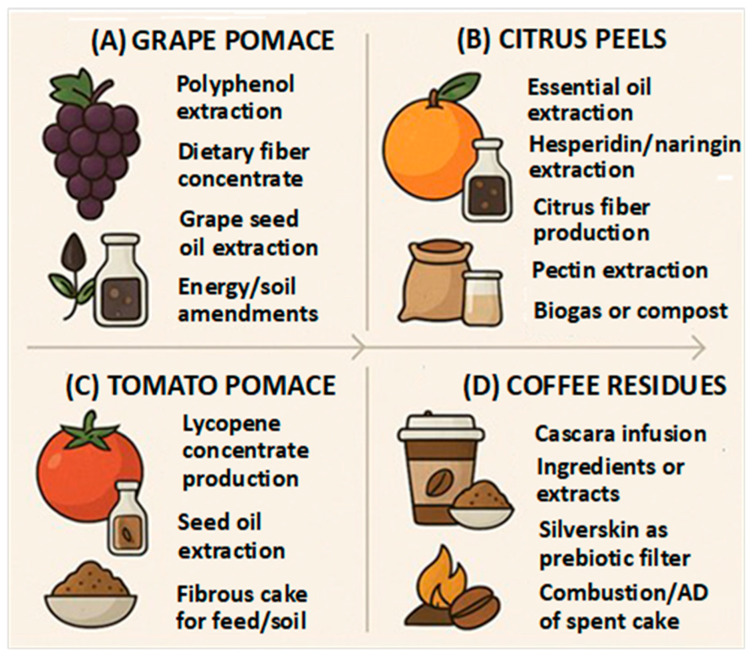
Cascading valorization of agri-food residues. (**A**): grape pomace; (**B**) citrus peels; (**C**) tomato pomace; (**D**) coffee residues.

Figure 1 illustrates how cascading strategies can be operationalized for representative residues, including grape pomace, citrus peels, tomato pomace, and coffee by-products, while highlighting the progressive extraction of high-value components followed by fiber, lipid, and energy streams.

LCA-aligned decision-making strengthens the sustainability of by-product recovery chains, guiding solvent choice, energy integration, and co-product utilization [105]. Recent regulatory advances, such as EFSA’s authorization of cascara as a novel food [106], demonstrate increasing institutional support for upcycled ingredients within circular bioeconomy frameworks. Together, these insights underscore that when guided by LCA, cascading biorefineries can transform agri-food residues into high-value ingredients, biomaterials, and energy carriers, supporting environmental goals while generating economic and nutritional benefits.

Tomato pomace, a byproduct of tomato processing, is a rich source of lycopene, seed oil, and fibrous material [107,108]. A cascading valorization approach for tomato pomace involves extracting lycopene for nutraceutical or food applications, recovering oil from the seeds, and reusing the remaining fibrous material as feed or soil amendment (Figure 1C).Coffee residues include cascara and silverskin, rich sources of bioactive compounds and fiber [109,110,111]. A cascading valorization approach for coffee residues could involve the following steps (Figure 1D): (i) cascara, the dried coffee cherry pulp, can be used to produce infusion ingredients or extracts; these products are rich in antioxidants and can be used in beverages and food products; (ii) silverskin, the thin layer of skin that covers the coffee bean, can be processed to produce a prebiotic fiber; this fiber can be used as a food ingredient to promote gut health; finally, (iii), the remaining spent coffee grounds can be used for energy production through combustion or anaerobic digestion.

Cascading valorization of agri-food residues offers a sustainable and economically viable approach to waste management. By sequentially extracting valuable components, this strategy minimizes waste, maximizes resource utilization, and promotes a circular economy. The examples provided demonstrate the potential of this approach for a variety of agri-food residues, highlighting the importance of developing innovative technologies and processes to unlock the full value of these resources.

## 10. Market Translation, Economic Drivers, and Policy Frameworks for Residue-Derived Bioactives

### 10.1. Introductory Considerations

The translation of bioactive compounds recovered from agri-food residues into commercial products requires an integrated approach that bridges extraction technology, formulation science, regulatory frameworks, and market strategy. The versatility of these compounds—including polyphenols, carotenoids, dietary fibers, and xanthones—enables their use as multifunctional ingredients across food, nutraceutical, cosmetic, and biomaterial sectors. Successful product design depends on maintaining bioactivity and stability during processing and storage, while ensuring safety, compliance, and consumer acceptance. Innovative delivery systems such as microencapsulation, nanoemulsions, and polymeric films have emerged to enhance bioavailability and controlled release, expanding their potential use across multiple industries.

From a circular-economy perspective, integrating bioactives into diverse product categories maximizes the overall value of agro-industrial streams and reduces waste generation [36]. The same residue may yield both functional molecules and structural materials, including fibers, pectins, proteins, or pigments, supporting a cascading biorefinery model that valorizes every fraction [112]. Recent techno-economic analyses reveal that integrating high-value product streams (e.g., phenolics or carotenoids) with lower-value co-products (fibers, lipids, feedstocks) significantly improves process profitability and shortens payback periods for residue-based biorefineries [113]. Additionally, emerging market research shows increasing demand for natural and “clean-label” ingredients sourced from upcycling processes, driven by consumer preference for sustainability and transparency [114].

Bioactive compounds recovered from food-processing by-products therefore offer broad applicability across industries (Table 5). However, their successful deployment in global markets depends on strong alignment between technological innovation, regulatory clarity, and supportive economic instruments.

### 10.2. Regulatory Footholds and Global Alignment

Regulatory approvals, including health claims, Novel Food authorizations, and GRAS notifications, provide essential pathways for de-risking commercial development. The EU’s approved health claim for olive-oil phenolics, particularly HT, is one of the most cited examples of successful residue-derived bioactive valorization [115]. Similarly, the EFSA granted Novel Food status to cascara (dried coffee cherry pulp), demonstrating regulatory openness to upcycled ingredients [116]. Regulatory convergence remains essential for global commercialization. Comparative analyses show that alignment between EFSA, FDA, and national agencies accelerates international trade and avoids duplicated toxicity testing requirements [117]. In the United States, GRAS notices for citrus fibers and botanical extracts streamline market entry and reduce approval timelines [118].

### 10.3. Eco-Labels, Upcycled Certification, and Consumer Perception

Upcycled certifications and eco-labels communicate sustainability value to consumers, driving acceptance of ingredients derived from innovative circular processes. Recent international surveys show that transparent labeling, clear sustainability narratives, and simplified messaging significantly increase purchase intent for upcycled foods [119].

Cross-country studies involving consumers in the United Kingdom, Denmark, Germany, Portugal, and Italy demonstrate that “upcycled,” “sustainable,” and “minimal waste” claims enhance perceived product quality and trust [120]. Meta-analytical evidence shows that environmental labels consistently improve consumers’ willingness to pay for sustainable foods, especially when environmental benefits are quantifiable and linked to recognized certification schemes [121]. These insights underline the strategic importance of eco-labeling for accelerating market uptake of residue-derived ingredients.

### 10.4. Public Procurement, Innovation Funding, and Bioeconomy Clusters

Public procurement and innovation funding programs are essential economic mechanisms that promote circular ingredients and reduce the capital expenditure associated with early-stage biorefineries. Analyses of the Bio-Based Industries Joint Undertaking (BBI-JU) and its successor, the Circular Bio-Based Europe Joint Undertaking (CBE-JU), demonstrate that such initiatives directly finance first-of-a-kind biorefineries and lower the risk profile of residue-based value chains [122]. Innovation funds strategically target pilot-scale and demonstration-scale facilities, enabling technological maturation (TRL increase) and facilitating industrial symbiosis models that integrate agriculture, food processing, and biotechnology sectors [122]. Public procurement schemes also create a demand-pull effect for sustainable products, particularly in institutional catering, school systems, hospitals, and public food services [123]. Regional bioeconomy clusters, often combining industry, academia, and local governments, are increasingly recognized as catalysts for technology transfer, skills development, and rural economic revitalization [124].

## 11. Research Gaps and Future Directions

### 11.1. Introdutory Considerations

Despite substantial advances in the valorization of agri-food residues and the deployment of green extraction technologies, several scientific and technological gaps limit large-scale implementation and the full exploitation of residue-derived bioactives. Addressing these gaps requires coordinated progress in extraction chemistry, process engineering, toxicology, human nutrition, and environmental assessment.

### 11.2. Need for Standardized Human Trials

Clinical evidence for residue-derived bioactives remains limited, with robust data primarily available for olive phenolics and citrus flavanones. Extracts from grape pomace, pomegranate peel, coffee silverskin, banana peel, and mango peel lack standardized human interventions. Future trials should employ chemically standardized extracts with defined markers (e.g., HT, hesperidin, punicalagin); evaluate dose–response relationships using validated cardiometabolic, inflammatory, and gut-related biomarkers; account for inter-individual metabolic variability, particularly for microbiota-derived metabolites such as urolithin A [125]. Such studies are essential to establish clinical relevance and ensure regulatory acceptance.

### 11.3. Harmonized LCA Methodologies for Bioactive Extraction

Life cycle assessment (LCA) has been increasingly applied to evaluate the environmental burdens of bioactive extraction; however, methodological heterogeneity limits comparability across studies. Key sources of divergence include system boundary definitions for cascading biorefineries; allocation rules for multi-output processes; assumptions regarding solvent recovery in MAE/UAE/SFE/NADES; integration of circularity metrics and avoided-burden credits. Recent analyses call for residue-specific LCA frameworks tailored to bioactive extraction processes [126]. Developing harmonized protocols will improve the accuracy of environmental benchmarking and guide technology selection.

### 11.4. Industrial Scalability of Green Extraction Systems

Green extraction technologies, NADES, EAE, MAE, and UAE, offer lower environmental impacts and improved selectivity compared to conventional methods. However, industrial deployment remains constrained by: high viscosity and complex recovery of NADES; limited enzyme specificity for heterogeneous plant matrices; insufficient development of continuous-flow MAE/UAE reactors; sparse techno-economic analyses under industrially realistic conditions. Emerging continuous-flow platforms and hybrid extraction systems show promise for industrial scale-up but require further validation [39].

### 11.5. Long-Term Safety, Bioavailability, and Microbiome Interactions

Data on chronic safety, bioavailability, and microbiome-mediated metabolism of residue-derived extracts remain scarce. Critical gaps involve: long-term toxicity and allergenicity assessments; influence of food matrices on phenolic bioaccessibility; metabolic diversity in gut-microbiota transformations, especially the conversion of ellagitannins to urolithins [127]; interactions between fibers and phenolic compounds and their impact on SCFA production and colonic phenolic metabolites [128]. Advanced omics, in vivo metabolic profiling, and controlled feeding studies will be critical to clarify these mechanisms. Future research must integrate standardized clinical protocols, uniform LCA frameworks, scalable extraction technologies, and comprehensive safety and microbiome assessments. Such progress will enable the transition of residue-derived bioactives from laboratory concepts to industrially viable and nutritionally effective components of the circular bioeconomy.

## 12. Commercial Deployment and Patent Landscape

### 12.1. General Aspects

The industrial translation of bioactive compounds recovered from agri-food residues has advanced rapidly over the past decade, reflecting scientific maturation, regulatory acceptance, and strong consumer-driven demand for sustainable ingredients. Improvements in green extraction (UAE, MAE, SFE, NADES), process intensification, and ingredient standardization have facilitated the transition from laboratory feasibility to scalable commercial operations. Companies across the food, nutraceutical, cosmetic, and biomaterials sectors are increasingly adopting upcycled ingredients, motivated by sustainability targets, regulatory incentives, and market differentiation opportunities.

Commercial deployment has been supported by a convergence of technological maturity, quality-assurance frameworks, and market trends favoring clean-label and plant-based products. The expansion of eco-certification programs, such as Upcycled Certified™, together with regulatory mechanisms including GRAS notifications and EFSA Novel Food approvals, has accelerated the integration of residue-derived bioactives into global value chains. Recent analyses show that sectors such as beverages, dietary supplements, functional foods, sports nutrition, and cosmetics are the fastest-growing segments for upcycled bioactive ingredients, with annual growth rates often exceeding 8–12% [129]. Below, representative examples illustrate how industrial stakeholders have successfully scaled the valorization of residue-derived bioactives.

### 12.2. Olive By-Products → Hydroxytyrosol (HT)

The EU recognizes a health claim for olive-oil phenolics (HT and derivatives) supporting protection against lipid oxidation at ≥5 mg·day^−1^ (Commission Regulation (EU) No 432/2012). This regulatory foothold has enabled the commercialization of standardized HT extracts from olive-mill wastewater and pomace. Companies such as Genosa^®^ and Olive Life^®^ market HT-rich formulations for nutraceuticals, beverages, and cosmetic applications. Recent studies confirm high antioxidant and anti-inflammatory efficacy of standardized HT ingredients in humans, reinforcing market expansion [70,130].

### 12.3. Brewer’s Spent Grain (BSG) → Proteins and Fibers

AB InBev’s EverGrain™ platform exemplifies large-scale valorization of BSG through protein isolation (EverPro™) and fiber concentrates certified by the Upcycled Food Association [131]. These ingredients provide environmentally favorable alternatives to conventional plant proteins, with LCA studies reporting 30–50% lower GHG emissions compared with soy-protein isolates [132].

### 12.4. Citrus Peels → Citrus Fiber and Flavanones

CP Kelco’s NUTRAVA™ Citrus Fiber, validated under GRAS Notice No. 943, is derived from juicing residues using physical processing with minimal solvents. In parallel, nutraceutical-grade hesperidin and naringin extracts are increasingly adopted in cardiometabolic health formulations [133]. This dual valorization, structural (fiber) and functional (flavanones), demonstrates circular product diversification.

### 12.5. Coffee Residues → Cascara and Silverskin

EFSA approved dried coffee husk (cascara) as a Novel Food in 2021, enabling its use in beverages and infusions [106]. Coffee silverskin is gaining traction as a prebiotic, antioxidant, and fiber-rich ingredient, supported by emerging human and in vitro data [134]. These valorization routes transform high-volume coffee-processing by-products into regulated functional materials.

### 12.6. Tomato Pomace/Peels → Lycopene Concentrates

Sustainable lycopene production using ethanol–terpene mixtures or supercritical CO_2_ has achieved commercial maturity [135,136]. Industrial manufacturers supply lycopene for food, cosmetics, and nutraceuticals, demonstrating high extraction selectivity and strong scalability under green-processing conditions.

### 12.7. Grape Seeds/Pomace → Polyphenol Concentrates

The MegaNatural^®^ product line (Polyphenolics Inc., Madera, CA, USA) offers standardized grape-seed extracts enriched in low-polymer procyanidins, supported by multiple patents and clinical studies demonstrating cardiovascular benefits [68]. This case exemplifies successful conversion of winery by-products into patented, high-value nutraceuticals.

### 12.8. Patent to Market Landscape

The patent landscape illustrates the transition from basic discovery to market-oriented innovation. Over the last decade, the number of patents related to residue-derived bioactives has expanded significantly, especially in the United States, the EU, China, and Japan. Patented innovations frequently target improved extraction selectivity, process sustainability, stabilization of active fractions, and multifunctional applications enabling cross-sector deployment. Representative patents include olive side streams (HT): US 7,713,569; WO 2004/005228; pomegranate peel: CN 105662946 A; EP 4 470 630 A1; grape seeds: US 8,075,929 B2; citrus peel flavanones: CN 103601775 A; WO 2015/006863; cocoa shells: US 2006/0269633 A1; US 8,603,547 B2; and tomato pomace: WO 2025/063836 A1.

The commercial success of upcycled bioactives depends on harmonized safety, labeling, and quality frameworks. Regulatory agencies such as EFSA, FDA, and ANVISA increasingly accept residue-derived ingredients when supported by toxicological and compositional data.

At the same time, the market landscape for sustainable ingredients continues to expand. Growing consumer demand for clean-label, minimally processed, and traceable products has driven rapid adoption of circular ingredients. Eco-labeling programs, transparent sourcing, and sustainability storytelling further enhance consumer trust and willingness to pay [137,138]. Together, these regulatory, technological, and market drivers reinforce the position of residue-derived bioactives as credible, high-value components of the circular bioeconomy.

## 13. New Perspectives in Research on Residue Valorization for Bioactive Recovery

In addition to the more conventional agro-industrial residues (pomaces, peels, bran, spent grains), emerging research is beginning to focus on novel and underexplored residue streams as sources of bioactives. For instance, agave bagasse from mezcal or tequila production has recently been shown to contain appreciable levels of phenolic compounds and dietary fiber, with marked seasonal variations in antioxidant capacity [139]. Similarly, agricultural glasshouse residues, such as stems, leaves, and trimmings from tomato [140,141] and pepper [142], and pruning wastes are being re-evaluated as potential matrices for extracting flavonoids, terpenoids, and plant hormones using green solvents [143]. In parallel, artificial intelligence and machine learning workflows are emerging to enable real-time monitoring, data-driven prediction of yield and selectivity, and algorithmic optimization of solvent systems and biocatalytic parameters, effectively digitizing residue valorization while improving efficiency and environmental performance [144]. Another frontier lies in food processing effluents, dilute aqueous streams containing phenolics, peptides, and oligosaccharides that typically escape conventional recovery routes. Advanced membrane separations (e.g., nanofiltration, electrodialysis), adsorbent materials with tailored affinity, and in situ concentration techniques may convert these “loss streams” into new sources of functional ingredients [145].

Apart from temperate residues, there is a growing body of work focusing on tropical and Brazilian biomasses traditionally underutilized but now recognized for their bioactive potential (Table 6). These include jabuticaba peel rich in anthocyanins [146]; cashew apple bagasse containing both phenolics and pectins; MAE of pectin from cupuassu pod husk; pequi peel and buriti shell flours as sources of phenolics; and açaí and acerola residues with remarkable antioxidant and antimicrobial properties. In parallel, mango peels from large-scale fruit processing chains have been valorized through sequential extraction of phenolics and pectin from mango peel assisted by ultrasound as models of green solvent intensification. Together, these studies highlight the emergence of tropical fruit residues as promising candidates for the next generation of circular-bioeconomy ingredients, particularly relevant for biodiverse regions such as Brazil, where agricultural residues are abundant yet still undervalued.

Another promising avenue involves harnessing microbial or fermentation residues (biomass, cell walls, spent media) from industrial biotechnology, which can yield bioactive peptides, exopolysaccharides, or phenolic conjugates. Reviews on plant-derived bioactive peptides emphasize that enzymatic hydrolysis of residual proteins from crop or fermentation sources releases peptides with antioxidant, antihypertensive, or antimicrobial activity, yet only a small proportion of valorization strategies target liquid or microbial residues, representing both a research gap and an industrial opportunity [166]. Recent studies have shown that spent microbial biomass—especially from *Saccharomyces*, *Lactobacillus*, and filamentous fungi—can yield postbiotic metabolites and cell-wall–derived polysaccharides with immunomodulatory and barrier-supporting functions, expanding the scope of residue-derived functional ingredients [167,168]. The integration of omics-guided screening, machine learning modeling, and process intensification could greatly accelerate the identification and recovery of high-value molecules from such unconventional feedstocks.

Finally, cross-domain valorization strategies are emerging that couple CO_2_ fermentative off-gas capture with microbial conversion into organic acids or phenolic precursors or integrate electro-bioreactor systems for upgrading residual streams into bioactive intermediates [169,170]. Recent bioprocess innovations demonstrate the feasibility of using carbon-fixing microbes (e.g., acetogens and hydrogenotrophs) to transform CO_2_ and syngas into metabolite pools suitable for downstream conversion into phenolic precursors and bioactive intermediates [171]. These systems of hybrid “residue-to-bioactive” blur the boundaries between waste valorization, biomanufacturing, and carbon circularity, defining a new paradigm for next-generation circular biorefineries tailored to both tropical and global contexts.

## 14. Concluding Remarks

Agri-food residues represent a consistent and scalable foundation for developing bioactive ingredients aligned with modern sustainability goals. Across diverse residue types and extraction approaches, evidence shows that these side streams can yield reproducible chemical profiles and biologically meaningful activities, supporting their use in functional and health-oriented applications. Recent progress in green processing and analytical traceability has expanded the feasibility of transforming these materials into standardized, high-quality products.

Advancing this field will require the systematic standardization of analytical and quality criteria, rigorous validation of biological effects through adequately powered and well-controlled human intervention studies, and the explicit incorporation of environmental and techno-economic metrics into process design and scale-up. Consolidating these pillars will facilitate the progression from experimental proof-of-concept findings to the development of robust, commercially viable ingredients produced from residue-derived value chains. Grounding future research efforts in methodological rigor and sustainability-driven frameworks will enable agri-food residues to assume a definitive role as renewable and strategically relevant sources of next-generation bioactive compounds.

## Figures and Tables

**Table 1:** Summary of main green extraction technologies applied to agri-food residues.

Extraction Technology	Working Principle/Main Bioactives Recovered	Operational Advantages	Limitations/Challenges
Ultrasound-Assisted Extraction (UAE)	Acoustic cavitation generates microbubble formation, collapse, and cell disruption/Polyphenols, phenolic acids, pigments	Low energy demand; short extraction times; compatible with aqueous–ethanolic solvents; effective as a pre-treatment	Limited scalability at industrial volumes; uneven cavitation in large reactors
Microwave-Assisted Extraction (MAE)	Dielectric heating induces rapid molecular rotation and internal heating, enhancing cell rupture/Polyphenols, flavonoids, alkaloids	Very fast extraction; higher yields; reduced solvent use; suitable for polar matrices	Non-uniform heating in batch systems requires matrices with dielectric properties
Pressurized Liquid Extraction (PLE/ASE)	High pressure + moderate temperature increase solvent diffusivity while keeping it in liquid state/Phenolic acids, flavonoids, carotenoids	High extraction efficiency; reproducible; >70% solvent reduction; suitable for multi-step biorefineries	Elevated temperatures may affect thermolabile compounds
Supercritical CO_2_ Extraction (SFE)	CO_2_ above its critical point offers gas-like diffusivity and liquid-like solvating power/Carotenoids, sterols, lipophilic phenolics	Solvent-free extracts; high purity; recyclable CO_2_; environmentally safe	High capital cost; lower efficiency for highly polar compounds unless co-solvents are added
Natural Deep Eutectic Solvents (NADES)	Hydrogen-bonded natural components form tunable, biodegradable solvents/Polyphenols, flavonoids, alkaloids	High selectivity; biodegradable; 20–50% higher yields than aqueous ethanol	High viscosity; solvent recovery still challenging
Enzyme-Assisted Extraction (EAE)	Hydrolytic enzymes degrade polysaccharide matrices and release bound compounds/Bound phenolics, ferulates, encapsulated bioactives	Mild temperatures; reduced chemical use; enhanced release of bound phenolics; synergistic with UAE/PLE	Enzyme cost; longer processing times

**Table 2:** In vitro evidence (2016–2025) for bioactives identified in agri-food residues with green extraction methods, intended applications, and circularity notes.

Chemical Class	Identified Bioactives	Residue (Origin) and Circularity	Green Extraction Method and In Vitro Findings	Intended Application Circularity/LCA Notes	Ref.
Polyphenols (anthocyanins, flavanols, phenolic acids)	Catechin, epicatechin, gallic and caffeic acids	Grape pomace (wine coproduct), reused as antimicrobial/antioxidant ingredient	Aqueous/ethanolic UAE. Antioxidant and antibacterial activity vs. pathogens; antibiofilm effects	Food preservative, antioxidant system. Valorizes winery waste; replaces synthetic antioxidants	[62]
Ellagitannins/phenolics	Punicalagin, punicalin, ellagic acid	Pomegranate peel (juice waste), anti-inflammatory extract	Hydroethanolic extraction/NADES Reduced TNF-α–driven inflammation in Caco-2 cells; antioxidant capacity	Nutraceutical, anti-inflammatory High phenolic yield; integrates with juice lines	[21]
Flavonols	Quercetin, kaempferol	Onion skins (processing waste), pigment recovery	Ethanol-based UAE or MAE Strong antioxidant and antimicrobial activity	Natural colorant and antioxidant Utilizes outer skins; replaces synthetic dyes	[40]
Bound and free phenolics (hydroxy-cinnamates)	Ferulic, p-coumaric, vanillic, syringic acids	Brewers’ spent grain (brewery by-product), food/cosmetic use	Enzymatic hydrolysis + UAE. Tyrosinase inhibition; antioxidant activity	Cosmetic antioxidant, food ingredient High-volume residue; enzyme-assisted recovery	[28]
Polyphenols and methylxanthines	Theobromine, caffeine, catechins	Cocoa bean shells (chocolate industry waste)	Hydroethanolic extraction. Antimicrobial vs. oral and foodborne pathogens; antioxidant effect	Oral-care, food antimicrobial Upcycles cocoa shells; reduces waste	[32]
Carotenoids and phenolics	Lycopene, β-carotene, rutin	Tomato pomace (canning waste)—natural colorant	Pulsed ultrasound-assisted. Antioxidant protection against lipid oxidation	Colorant and antioxidant for foods Valorizes tomato residues; circular pigment source	[63]
Polyphenols Catechin	Epigalocatequina-3-galato, epicocatequina-3-galato, epigalocatequina, and epicocatequina	Tea pomace (spent leaves), nano-delivery system	Aqueous ethanolic extraction. Retained antioxidant activity in packaging composites	Active packaging, antioxidant release Reduces plastic waste; biodegradable systems	[64]
Polyphenols (apple)	Chlorogenic acid, phloridzin, quercetin	Apple pomace (juice residue), anti-inflammatory source	Cold Press-assisted solvent extraction. Antioxidant, anti-inflammatory, anti-thrombotic effects	Functional food ingredient Combined with pectin recovery	[65]
Coffee by-product fibers and phenolics	Chlorogenic acids, xylans	Coffee silverskin (roasting by-product)	Green UAE. Antioxidant and prebiotic activity; supports probiotics	Prebiotic ingredient Enables circular coffee chain	[66]
Fibers and phenolics	Insoluble fiber-bound polyphenols	Rice bran (milling residue)	Enzymatic and aqueous extraction. Antioxidant and lipid-peroxidation inhibition	Functional fiber ingredient Promotes zero-waste rice valorization	[67]

**Table 4:** In vivo evidence (2016–2025) in humans for bioactives from agri-food residues, with models, principal outcomes, green extraction, intended application, and circularity notes.

Chemical Class/Identified Bioactives	Residue Origin and Circularity/LCA Notes/Green Extraction	In Vivo Model and Outcomes/Intended Application	Ref.
Simple phenols (olive by-products). Hydroxytyrosol (HT)	Olive pomace/extra virgin olive oil side streams—purified HT. Adds value to olive pomace; aligns with European Union health claim context. Phenolics purified from side streams	Randomized controlled trial (RCT) (overweight/prediabetes): improved antioxidant/anti-inflammatory status Clinical-grade nutraceutical	[70]
Fibers and phenolics Insoluble fiber-bound polyphenols	Rice bran (milling residue) Enzymatic and aqueous extraction	Antioxidant and lipid-peroxidation inhibition Functional fiber ingredient Promotes zero-waste rice valorization	[67]
Citrus flavanones. Hesperidin, naringin	Citrus peels (juice industry)—standardized flavanones. Valorization with essential oils/pectin co-streams. Hydroethanolic extraction; standardization	Human RCTs/meta-analysis: improved lipids, blood pressure, and inflammatory markers. Cardiometabolic support	[71]
Maltodextrinated grape pomace extract	Randomized controlled clinical trial (99 patients) with grape pomace extract for diabetic retinopathy.	The grape pomace extract group showed improvement in best-corrected visual acuity	[85]
Grape pomace polyphenols (29.6%)	Randomized cross-over clinical trial was conducted (49 patients exhibiting at least two metabolic syndrome factors were supplemented with a daily dose of 8 g for 6 weeks, with an equivalent control (CTL) period.	The reduction in insulin levels in subjects at cardiometabolic risk upon grape pomace supplementation appears not to be induced by changes in the major subgroups of gut microbiota.	[84]

**Table 5:** Bioactives derived from food processing by-products are useful in industrial areas.

Industrial Areas	Bioactives Derived from Food Processing by-Products
Food and beverages	Extracts can serve as natural antioxidants, color stabilizers (e.g., lycopene from tomato pomace, anthocyanins from berry pomace), texturizers (e.g., citrus fiber, pectins from fruit peels), and prebiotic ingredients (e.g., silverskin from coffee, bran from grains)
Nutraceuticals and medical foods	Standardized extracts with specified marker compound concentrations (e.g., punicalagin in pomegranate extract, hesperidin in citrus extract, hydroxytyrosol in olive extract) can be used in nutraceutical formulations and medical foods
Cosmetics and personal care	Anti-oxidative and anti-inflammatory actives from sources like pomegranate peel, olive pomace, grape pomace, and cocoa shells can be incorporated into cosmetic and personal care products. Cocoa shell polyphenols can also be used as oral-care antimicrobials
Active packaging and biomaterials	Catechins from tea pomace and phenolic compounds from fruit peels can be embedded in biodegradable films for controlled release and oxidation control in active packaging applications

**Table 6:** Emerging Brazilian residue streams for bioactive recovery.

Residue/Waste Stream	Bioactive(s)/Functional Target	Key Findings/Highlights	Ref.
Jabuticaba (*Plinia peruviana* (Poir.) Govaerts) peel	Anthocyanins, total phenolics; antioxidant	Optimized probe-UAE and concentration of phenolics by up to 45%; identified cyanidin-3-glucoside and related anthocyanins.	[147]
Jabuticaba [*Myrciaria jaboticaba* (Vell.) O. Berg.] peel	Anthocyanins	The jabuticaba peel extract inhibited starch and very strongly triglyceride absorption in mice.	[148]
Jabuticaba (*Myrciaria/Plinia jaboticaba*). peel (and bagasse/seed in some works)	Anthocyanins (e.g., cyanidin-3-O-glucoside), ellagitannins, phenolic acids	Conventional solvent extraction; comparative seasonal profiling; nanoencapsulation in phospholipid vesicles Antioxidant capacity; in vitro anti-cancer/keratinocyte oxidative stress models; techno-functional flours	[146]
Cashew (*Anacardium occidentale* L.) apple bagasse	Phenolics, food-grade pectin	Hydrothermal/pressurized water processes enable simultaneous valorization (phenolics + pectin) from cashew apple bagasse; scalable route indicated.	[149]
Cupuaçu [*Theobroma grandiflorum* (Willd. ex Spreng.) Schum.]	Pectin	Microwave-assisted extraction of pectin from cupuaçu pod husk	[150]
Pequi (*Caryocar brasiliense* Camb.) peel	Phenolics; antioxidant	Characterization confirmed high phenolic content in pequi peel extracts; supports residue as source of functional ingredients	[151]
Buriti (*Mauritia flexuosa* L.) shell flour	Phenolics	Shell flour showed relevant phytochemicals and antioxidant potential; proposes waste-to-ingredient pathway for native Brazilian fruit residues.	[152]
Buriti (*Mauritia flexuosa*) shell flour	Carotenoids	High content of carotenoids, mainly β-carotene (27.18–62.94 µg/100 g) and α-carotene (18.23–60.28 µg/100 g)	[152]
Açaí (*Euterpe oleracea* Mart.) seeds (roasted)—upcycled beverage	Phenolics (e.g., chlorogenic acids, procyanidins); functional beverage	Roasted açaí seed proposed as caffeine-free “coffee” with characterized phenolics; illustrates food-grade upcycling of a major residue stream	[153]
Juçara (*Euterpe edulis* Mart.) fruit by-products	Phenolics, antioxidant, and antibacterial potential	The peel flour presented antioxidant and antibacterial potentials	[154]
Acerola (*Malpighia emarginata* DC) by-products	Phenolics; antioxidant and antibacterial	Probe-UAE optimized by Box–Behnken design increased total phenolic compounds recovery/antioxidant activity; antibacterial activity validated	[155]
Mango peel (*Mangifera indica* L.)	Phenolics and terpenoids; green solvent extraction	NADES-based UAE demonstrated as a green/efficient route for phenolics/terpenoids from peel.	[156]
Mango peel (*Mangifera indica* L.)	Phenolics and pectin	Sequential extraction of phenolics and pectin from mango peel assisted by ultrasound.	[157]
Baru (*Dipteryx alata* Vogel). peel/pulp/endocarp (under-used parts)	Diverse phenolics by Paper Spray Mass Spectrometry fingerprinting	Paper-spray mass spectrometry (profiling) + conventional extracts. Chemical mapping to support residue valorization.	[158]
Cagaita (*Eugenia dysenterica* DC) Peel + seed (by-products)	Catechin, epicatechin, quercetin (High-Performance Liquid Chromatography with Diode Array Detection), total phenolics	UAE was optimized by Response Surface Methodology. Antioxidant, antimicrobial, antibiofilm activities.	[159]
Buriti (*Mauritia Flexuosa* L.) Peel and pulp (including shell)	Carotenoids (β-carotene), phenolic compounds	Eco-friendly supramolecular solvents (octanoic-acid based) and ethanol, also with PLE. Antioxidant capacity; antibacterial/modulatory effects reported for extracts.	[160]
Bacaba (*Oenocarpus bacaba* Mart./*Oenocarpus distichus* Mart.). peel/residue	Anthocyanins, rutin, epicatechin; high total phenolics	Optimized solvent extraction; compositional profiling. High antioxidant capacity in residues; food prototype uses (e.g., beverages).	[161]
Guabiroba (*Campomanesia xanthocarpa* O. Berg.). Peel extract used as additive	Phenolics (tannins, flavonoids)	Hydroalcoholic extraction; incorporation study. Natural antioxidant/clean-label preservative in tilapia pâté.	[162]
Purple araçá (*Psidium myrtoides* O. Berg.) by-products (peel/seed)	Phenolic acids and flavonoids	NADES + UAE (green extraction) Antioxidant and in vitro antidiabetic activity	[49]
Camu-camu [*Myrciaria dubia* (Kunth) McVaugh] peel and seed (industrial by-residues)	Ellagic acid derivatives, procyanidins, other phenolics	Conventional/optimized extractions; response-surface optimization for seed coat. Antioxidant; anti-diabetic/anti-hypertensive/antiproliferative in vitro; food fortification.	[163]
Açaí (*Euterpe oleracea* Mart.) Seeds (major processing waste)	Procyanidins (B type), catechin/epicatechin	PLE; tech-economic assessed. Antioxidant extracts; feasibility for industrial recovery.	[164]
Umbu (*Spondias tuberosa* Arruda) peel	Phenolics (Ultra-Performance Liquid Chromatography/Quadrupole Time-of-Flight Mass Spectrometry)	Thermal-assisted solid–liquid extraction optimized by Response Surface Methodology. Antioxidant, antimicrobial; α-amylase inhibition.	[165]

## Abbreviations

The following abbreviations are used in this manuscript:

ANVISA	Brazil’s National Health Surveillance Agency
BSG	Brewers’ spent grain
EAE	Enzyme-assisted extraction
EFSA	European Food Safety Authority
EU	European Union
FDA	U.S. Food and Drug Administration
GRAS	Generally Recognized as Safe
HT	Hydroxytyrosol
LCA	Life cycle assessment
MAE	Microwave-assisted extraction
NADES	Natural deep eutectic solvents
PLE	Pressurized liquid extraction
RCT	Randomized controlled trial
SCFAs	Short-chain fatty acids
SFE	Supercritical CO_2_ extraction
UAE	Ultrasound-assisted extraction
